# *Anaplasma phagocytophilum*—a widespread multi-host pathogen with highly adaptive strategies

**DOI:** 10.3389/fcimb.2013.00031

**Published:** 2013-07-22

**Authors:** Snorre Stuen, Erik G. Granquist, Cornelia Silaghi

**Affiliations:** ^1^Department of Production Animal Clinical Sciences, Norwegian School of Veterinary ScienceSandnes, Norway; ^2^Department of Production Animal Clinical Sciences, Norwegian School of Veterinary ScienceOslo, Norway; ^3^Department of Veterinärwissenschaftliches, Comparative Tropical Medicine and Parasitology, Ludwig-Maximilians-Universität MünchenMunich, Germany

**Keywords:** *Anaplasma phagocytophilum*, ecology, epidemiology, distribution, hosts, vectors

## Abstract

The bacterium *Anaplasma phagocytophilum* has for decades been known to cause the disease tick-borne fever (TBF) in domestic ruminants in *Ixodes ricinus*-infested areas in northern Europe. In recent years, the bacterium has been found associated with *Ixodes-tick* species more or less worldwide on the northern hemisphere. *A. phagocytophilum* has a broad host range and may cause severe disease in several mammalian species, including humans. However, the clinical symptoms vary from subclinical to fatal conditions, and considerable underreporting of clinical incidents is suspected in both human and veterinary medicine. Several variants of *A. phagocytophilum* have been genetically characterized. Identification and stratification into phylogenetic subfamilies has been based on cell culturing, experimental infections, PCR, and sequencing techniques. However, few genome sequences have been completed so far, thus observations on biological, ecological, and pathological differences between genotypes of the bacterium, have yet to be elucidated by molecular and experimental infection studies. The natural transmission cycles of various *A. phagocytophilum* variants, the involvement of their respective hosts and vectors involved, in particular the zoonotic potential, have to be unraveled. *A. phagocytophilum* is able to persist between seasons of tick activity in several mammalian species and movement of hosts and infected ticks on migrating animals or birds may spread the bacterium. In the present review, we focus on the ecology and epidemiology of *A. phagocytophilum*, especially the role of wildlife in contribution to the spread and sustainability of the infection in domestic livestock and humans.

## Introduction

The bacterium *Anaplasma phagocytophilum* has been known to cause disease in domestic ruminants (Europe) (Foggie, 1951) and horses (USA) (Gribble, 1969) for decades. More recently, the infection has been detected in several mammalian species, including humans, in areas on the northern hemisphere with endemic occurrence of *Ixodes* ticks. *A. phagocytophilum* as a bacterial species appears to be a generalist, infecting a wide range of animals. Multiple genetic variants of the bacterium have been characterized (Scharf et al., 2011) and subpopulations within the species are now being discussed. In this review, we present updated information especially concerning the ecology and epidemiology of *A. phagocytophilum*.

## History

During an experimental study on louping-ill (LI) in Scotland last century, some sheep contracted an unknown fever reaction on tick-infested pastures. The fever reaction was transmitted to other sheep by blood inoculation, but gave no protection against a later LI-virus infection. The disease was given the provisional name “tick-borne fever” (TBF), and the responsible pathogen was assumed to belong to the class *Rickettsia* (Gordon et al., 1932, 1940). The name TBF is still used for the infection in domestic ruminants in Europe. Anecdotally it could be mentioned that the Norwegian synonym of TBF is “sjodogg,” and this name was already used to describe a devastating illness in ruminants as early as year 1780 in a coastal area of western Norway (Stuen, 2003).

The causative agent of TBF was first classified as *Rickettsia phagocytophila* (Foggie, 1951). However, due to morphological resemblance with *Cytoecetes microti*, an organism found in the polymorphonuclear cells of the vole *Microtus pennsylvanicus* (Tyzzer, 1938), it was later suggested to include the TBF agent in the genus *Cytoecetes* in the tribe *Ehrlichia*, as *C. phagocytophila* (Foggie, 1962).

In 1974, the organism was named *Ehrlichia phagocytophila* in Bergey's manual of determinative bacteriology (Philip, 1974). The discovery of *E. chaffeensis* in 1986, causative agent of human monocytic ehrlichiosis (Maeda et al., 1987; Anderson et al., 1991), and the agent of human granulocytic ehrlichiosis (HGE) in 1994 (Bakken et al., 1994; Chen et al., 1994), initiated new studies on the host associations, epidemiology and taxonomy of the granulocytic *Ehrlichiae* (Ogden et al., 1998). Genus *Ehrlichia* was divided into three genogroups, of which the granulocytic group contained *E. phagocytophilum*, *E. equi* [described in horses (Gribble, 1969)] and the agent causing HGE. Later, a reclassification of the genus *Ehrlichia* was proposed, and based on phylogenetic studies, the granulocytic *Ehrlichia* group was renamed *Anaplasma phagocytophilum* (Dumler et al., 2001; Anonymous, 2002) (Table 1). However, it is still argued, whether the granulocytic *Anaplasma* should eventually be reclassified as distinct from the erythrocytic *Anaplasma* and returned to the previously published genus, *Cytoecetes* (Brouqui and Matsumoto, 2007).

**Table 1:** **Classification of genus *Anaplasma*, *Ehrlichia*, and *Neorickettsia* in the family *Anaplasmataceae* (modified after Dumler et al., 2001)**.

	**Genus**		
	***Anaplasma***	***Ehrlichia***	***Neorickettsia***
Species	*A. marginale*	*E. canis*	*N. risticii*
	*A. bovis*	*E. chaffeensis*	*N. sennetsu*
	*A. ovis*	*E. ewingii*	
	*A. phagocytophilum*	*E. muris*	
	*A. platys*	*E. ruminantium*	

## Clinical characteristics

Natural infection with *A. phagocytophilum* has been reported, as already mentioned, in humans and a variety of domestic and wild animal species (Foley et al., 1999), whereas fatal cases have so far only been reported in sheep, cattle, horses, reindeer, roe deer, moose, dogs, and humans (Jenkins et al., 2001; Stuen, 2003; Franzén et al., 2007; Heine et al., 2007).

The main disease problems associated with TBF in ruminants are seen in young animals, and individuals purchased from tick-free areas and placed on tick-infested pastures for the first time. The most characteristic symptoms in domestic ruminants are high fever, anorexia, dullness, and sudden drop in milk yield (Tuomi, 1967a). However, the fever reaction may vary according to the age of the animals, the variant of *A. phagocytophilum* involved, the host species and immunological status of the host (Foggie, 1951; Tuomi, 1967b; Woldehiwet and Scott, 1993; Stuen et al., 1998). Abortion in ewes and reduced fertility in rams have also been reported. In addition, reduced weight gain in *A. phagocytophilum* infected bullocks and lambs have been observed (Taylor and Kenny, 1980; Stuen et al., 1992; Grøva et al., 2011).

A variable degree of clinical symptoms have also been detected in other mammals, such as fever, anorexia, depression, apathy, distal edema, reluctance to move, and petechial bleedings in horses, while the symptoms in dogs are characterized by fever, depression, lameness, and anorexia. In cats the predominant signs are anorexia, lethargy, hyperesthesia, conjunctivitis, myalgia, arthralgia, lameness, and incoordination (Egenvall et al., 1997; Bjöersdorff et al., 1999; Cohn, 2003; Franzén et al., 2005; Heikkilä et al., 2010).

In humans, clinical manifestations range from mild self-limiting febrile illness, to fatal infections. Commonly, patients express non-specific influenza-like symptoms with fever, headache, myalgias, and malaise (Bakken et al., 1994; Dumler, 1996). In addition, thrombocytopenia, leukopenia, anemia, and increased aspartate and alanine aminotransferase activity in sera are reported (Bakken and Dumler, 2008). However, most human infections probably result in minimal or no clinical manifestations. Reports from the US, indicate a hospitalization rate of 36%, of which 7% need intensive care, while the case fatality rate is less than 1% (Dumler, 2012). A recent cohort study from China however, describes a mortality of 26.5% (22/83) in hospitalized patients (Li et al., 2011).

## Diagnostic and laboratory methods

### Clinical signs

Clinical signs in ruminants may be sudden onset of high fever (>41°C) and drop in milk yield, while symptoms in horses, dogs, and cats may be more vague and unspecific. In humans, a flu-like symptom 2–3 weeks after tick exposure is an indicator of infection. However, laboratory confirmation is required to verify the diagnosis (Woldehiwet, 2010). To our knowledge, chronic infection has not yet been confirmed in any host, although persistent infections have been found to occur in several mammalian species.

### Direct identification

Light microscopy of blood smears taken in the initial fever period is normally sufficient to state the diagnosis. Stained with May-Grünwald Giemsa, the organisms appear as blue cytoplasmic inclusions in monocytes and granular leucocytes, especially neutrophils (Foggie, 1951). Electron microscopy may also confirm the diagnosis of acute *Anaplasma* infection in blood or organs. Single or multiple organisms are then identified in clearly defined cytoplasmic vacuoles (Tuomi and von Bonsdorff, 1966; Rikihisa, 1991). Immuno-histochemistry on tissue samples could also be performed to confirm the diagnosis (Lepidi et al., 2000).

### Polymerase chain reaction (PCR) and cultivation

Several PCR techniques (conventional, nested, and real-time) for the identification of *A. phagocytophilum* infection in blood and tissue samples have been established primarily on basis of the *16S rRNA*, *groEL*, and *p44* genes (Chen et al., 1994; Courtney et al., 2004; Alberti et al., 2005a). Multiple variants of *A. phagocytophilum* have been genetically characterized. Identification and stratification into phylogenetic subfamilies have been based on cell culturing, experimental infections, PCR and sequencing techniques (Dumler et al., 2007). Cultivation of *A. phagocytophilum* in cell cultures has been described for variants isolated from human, dog, horse, roe deer, and sheep (Goodman et al., 1996; Munderloh et al., 1999; Bjöersdorff et al., 2002; Woldehiwet et al., 2002; Silaghi et al., 2011c).

### Serology

The presence of specific antibodies may support the diagnosis. A complement fixation test, counter-current immunoelectrophoresis test and an indirect immunofluorescent antibody (IFA) test can be used (Webster and Mitchell, 1988; Paxton and Scott, 1989). Several ELISA tests have also been developed (Ravyn et al., 1998; Magnarelli et al., 2001; Alleman et al., 2006; Woldehiwet and Yavari, 2012). A SNAP®4Dx® ELISA test is commercially available for rapid in-house identification of *A. phagocytophilum* antibodies in dog serum, but the kit has also been used successfully on horse and sheep sera (Granquist et al., 2010a; Hansen et al., 2010).

### Pathology

An enlarged spleen, up to 4–5 times the normal size with subcapsular bleedings, has for decades been regarded as indicative of TBF in sheep (Gordon et al., 1932; Øverås et al., 1993). No other typical pathological changes have been described (Munro et al., 1982; Campbell et al., 1994; Lepidi et al., 2000). An enlarged spleen with subcapsular bleedings has also been observed in roe deer and reindeer (Stuen, 2003).

Relative sensitivity of the diagnostic tests used for laboratory diagnostic confirmation of *A. phagocytophilum* infection in humans is shown in Table 2.

**Table 2:** **Relative sensitivity of diagnostic tests for *A. phagocytophilum* infection in humans (modified after Bakken and Dumler, 2006)**.

**Duration of illness (days)**	**Blood smear microscopy**	**HL-60 cell culture**	**PCR**	**IFAT**
0–7	Medium	Medium	High	Low
8–14	Low	Low	Low	Medium
15–30			Low	High
31–60				High
>60				High

## Treatment, prevention, and control

The drug of choice is tetracycline (Woldehiwet and Scott, 1993; Dumler, 1996). Doxycyclin hyclate, given orally or intravenously, has been effective in treating clinical cases of human granulocytic anaplasmosis, and has led to clinical improvement in 24–48 h. In human patients, treated with doxycycline for 7–10 days, infections have resolved completely and relapses have never been reported. In patients at risk of adverse drug reactions, rifampin therapy should be considered (Bakken and Dumler, 2006).

Current disease prevention strategies in domestic animals are based on the reduction of tick infestation by chemical acaricides, for instance at turn out on tick pasture. This is mostly done be dipping or with a variety of pour-on applications (Woldehiwet and Scott, 1993; Stuen, 2003). This treatment has to be repeated during the tick season. In the UK, long-acting tetracycline has also been used as a prophylactic measure given before animals are moved from tick-free environment into tick-infested pasture (Brodie et al., 1986; Woldehiwet, 2007). However, there is a growing concern about the environmental safety and human health, increasing costs of chemical control and the increasing resistance of ticks to pesticides (Samish et al., 2004).

Biological tick control is becoming an attractive approach to tick management. Biological control of tick infestations has been difficult because ticks have few natural enemies. Studies so far have concentrated of bacteria, entomopathogenic fungi, and nematodes (Samish et al., 2004). However, the main challenge is to create a sustainable biological control of ticks in the natural habitat.

Vaccines against *A. phagocytophilum* are not yet available. Several vaccine candidates have been suggested, but the development of an effective vaccine has so far been difficult (Ijdo et al., 1998; Herron et al., 2000; Ge and Rikihisa, 2006). In order to develop a vaccine, one challenge is to choose antigens that are conserved among all variants of *A. phagocytophilum*.

Vaccines against ticks are also an alternative option. The development of vaccines that target both ticks and pathogen transmission may provide a mean of controlling tick-borne infections through immunization of the human and animal population at risk or by immunization of the mammalian reservoir to minimize pathogen transmission (de la Fuente and Kocan, 2006). Gut-, salivary-, or cement antigen vaccines (recombinant Bm/Ba 86, Bm91, and 64TRP) have been tested, and TickGUARDPLUS and Gavac (both recombinant Bm86) are examples of commercially available vaccines from the early 1990's (Willardsen, 2004; Labuda et al., 2006; de la Fuente et al., 2007; Canales et al., 2009). Other vaccines that inhibit subolesin expression are now being tested. These vaccines cause degeneration of gut, salivary gland, reproductive -and embryonic tissues and causes sterility in male ticks (de la Fuente et al., 2006a,b,c). Tick vaccines are feasible control methods, cost-effective and environmentally friendly compared to chemical control (de la Fuente and Kocan, 2006).

## Transmission and colonization

*A. phagocytophilum* has, as its name implies, a partiality to phagocytic cells and is one of very few bacteria known to survive and replicate within neutrophil granulocytes (Choi et al., 2005). During tick feeding, neutrophil-associated-inflammatory-responses are modulated by various stimuli deployed by the tick sialome components (Beaufays et al., 2008; Guo et al., 2009; Heinze et al., 2012). Orchestration of vector—and bacterial interactions with the defensive mechanisms of the host animal seem to promote infection and transmission rather than controlling it, resulting in increased availability of infected cells in the circulating blood and at the site of tick bite (Choi et al., 2003, 2004; Granquist et al., 2010b; Chen et al., 2012). The low level of circulating organisms, detected between periods of bacteremia (Granquist et al., 2010c), may indicate temporary clearance of infected cells, possible margination of infected granulocytes to endothelial surface or immunologically modified intervals in generations of antigenically different organisms (Bakken et al., 1994; Beninati et al., 2006; Granquist et al., 2008). Because of the short-lived nature of circulating neutrophils, the role of these cells in establishing and maintaining infection has been questioned (Herron et al., 2005), however to date little is known about alternative cellular components involved in the invasion and colonization of *A. phagocytophilum* in the host organism (Granick et al., 2008).

*A. phagocytophilum* modulates the distribution of potential host cells and infected neutrophils, by inducing cytokine secretion and their receptors (Akkoyunlu et al., 2001; Scorpio et al., 2004) and promoting the loss of CD162 and CD62L (Choi et al., 2003). The bacterium further interacts with host cell ligands (Park et al., 2003; Granick et al., 2008), by surface exposed proteins known as adhesins (Yago et al., 2003; Ojogun et al., 2012) in order to facilitate internalization in the host cell (Wang et al., 2006).

The translocation of bacteria to the inside of host cells is receptor mediated and depending on transglutaminase activity (reviewed by Rikihisa, 2003). However, host cell specific differences to receptors and their components as well as their importance in the infection process seem to exist, which may explain why certain bacterial strains, e.g., ruminant *Ap* Variant 1 strain, are refractory to culture in commercially available cell lines (like the HL-60 cell line) (Carlyon et al., 2003; Herron et al., 2005; Reneer et al., 2006, 2008; Massung et al., 2007). Previous reports have shown that various tissues and cells are susceptible to infection by *A. phagocytophilum* (Klein et al., 1997; Munderloh et al., 2004). It has been shown that intravascular myeloid cells (mature) have a higher infection rate than cells located in the bone marrow which may indicate that precursor stages of myeloid cells express ligands different from mature neutrophils, thus being more refractory to binding and internalization of the organism (Bayard-Mc Neeley et al., 2004). The coincidence that *A. phagocytophilum* uses CD162 when infecting neutrophils, led to the hypothesis that endothelium may have a function in the pathogenesis of *A. phagocytophilum* infection *in vivo* (Herron et al., 2005). However, a field study of skin biopsies in sheep observed *A. phagocytophilum* in inflammatory cell infiltrates comprised of PMNs and macrophages in the dermis and subcutis, and occasionally restricted to the mid- and peripheral parts of the blood vessel walls during tick attachment, thus questioning the role of endothelium in the pathogenesis of *A. phagocytophilum* infection in in the earliest phases of tick bite inoculation (Granquist et al., 2010b). Interestingly A. *phagocytophilum* has the ability to delay host cell apoptosis by activation of an anti-apoptosis cascade (Sarkar et al., 2012). This is critical for intracellular survival and reproduction of *A. phagocytophilum* in the normally short lived neutrophil granulocytes (Yoshiie et al., 2000; Lee and Goodman, 2006). Unlike other Gram-negative bacteria, *A. phagocytophilum* lacks lipopolysaccharides and peptidoglycans, but compensates for the loss of membrane integrity by incorporation of cholesterol which allows the escape of Nod Like Receptor and Toll Like Receptor activation pathways to successfully infect vertebrate immune cells (Lin and Rikihisa, 2003a,b; Hotopp et al., 2006; Xiong et al., 2007). However, recent studies in mice have surprisingly shown that alternative pathways involving the Nod 1 and 2 associated receptor interacting protein 2 may be important in control and clearance of *A. phagocytophilum* infection (Sukumaran et al., 2012).

## Persistence

*A. phagocytophilum* has been found to persist in several mammalian hosts, such as sheep, dog, cattle, horses, and red deer (Foggie, 1951; Egenvall et al., 2000; Stuen, 2003; Larson et al., 2006; Franzén et al., 2009). However, this may vary according to the variants of the bacterium involved.

The ability of *A. phagocytophilum* to persist in immune-competent hosts between seasons of tick activity is a complex and coordinated interaction that through evolutionary steps, have left the genomes of *A. phagocytophilum* and related organisms, heavily reduced to comprise essential genes allowing for nearly infinite numbers of recombined antigens and macromolecular exchange with its host cell (Rikihisa, 2011; Rejmanek et al., 2012).

Cyclic bacteremias display as periodic peaks containing genetically distinct variants of major surface proteins (MSP) (Granquist et al., 2008, 2010a). The capacity to generate novel antigens when other organisms are already present (superinfection) results in persistence and maintenance of the organism in natural transmission cycles and possibly allows spatial spread in nature (Barbet et al., 2003; Rodriguez et al., 2005; Futse et al., 2008; Ladbury et al., 2008; Stuen et al., 2009). Variants of MSPs such as MSp2 (or P44) contain epitopes recognized by antibodies appearing subsequently, but not prior to the respective peaks of rickettsemia in which they are expressed (Barbet et al., 2003; Granquist et al., 2010c), indicating a true process of antigenic variation influenced by the host immune response. Sequence variation may be achieved by segmental gene conversion of a single polycistronic expression site by insertion of total or partial pseudogene sequences (Barbet et al., 2000; Granquist et al., 2008) with the possible formation of mosaics or chimeras (Rejmanek et al., 2012). The large repertoire of donor sequences in *A. phagocytophilum* suggests that this bacterium may however only require simple gene conversion to evade host immune surveillance (Lin et al., 2003). On the other hand, the close proximity of the partial recombinase gene, *recA*, which is commonly involved in homologous recombinations supports the theory that recombination of pseudogenes by insertion in the expression site occurs (Barbet et al., 2003; Lin et al., 2003).

## Vectors and competent vectors of *A. phagocytophilum*

*A. phagocytophilum* is transmitted by hard ticks of the *I. persulcatus*-complex. The main vector in Europe is *I. ricinus* (commonly known as sheep tick or castor been tick); in the Eastern US *I. scapularis* (deer tick or black-legged tick); in the Western US *I. pacificus* (Western black-legged tick), and in Asia *I. persulcatus* (taiga tick) (Woldehiwet, 2010). Vector competence has been proven for the American tick species *I. scapularis* (previously *I. dammini*), *I. pacificus*, and *I. spinipalpis* (Telford et al., 1996; Des Vignes et al., 1999; Zeidner et al., 2000; Teglas and Foley, 2006). Transovarial transmission has not been proven in *Ixodes* species, but in *Dermacentor albipictus*, which lifecycle involves a single host animal, representing a distinct ecological niche (Baldridge et al., 2009). As to current knowledge, a vertebrate reservoir host is necessary in nature for keeping the endemic cycle.

Prevalence data on molecular detection of *A. phagocytophilum* in questing ticks, show great variations within countries or continents where such studies have been performed. The infection rate in *I. scapularis* ranges from <1% up to 50% and in *I. pacificus* from <1% up to ~10% in the US. Additionally, *A. phagocytophilum* has been detected in questing *I. dentatus*, *Amblyomma americanum*, *Dermacentor variabilis*, and *D. occidentalis* (Table 4; Goethert and Telford, 2003). In Asia, detection rates varied in *I. persulcatus* between <1% up to 21.6% and questing *I. ovatus*, *I. nipponensis*, *D. silvarum*, *Haemaphysalis megaspinosa*, *H. douglasii*, *H. longicornis*, and *H. japonica* also contained DNA of *A. phagocytophilum* (Table 5). The greatest number of studies has been performed on questing *I. ricinus* ticks in Europe, where the prevalence rates vary between and also within countries. On average, the *A. phagocytophilum*-prevalence in *I. ricinus* in Europe ranges between <1% and ~20%, in *I. persulcatus*-endemic areas in Eastern Europe between 1.7 and 16.7%, and additionally DNA of *A. phagocytophilum* has been detected in questing *D. reticulatus*, *H. concinna*, and *I. ventalloi* (Table 3). Detailed information on worldwide prevalence rates of *A. phagocytophilum* in unfed ticks from the vegetation can be found in Tables 3–5.

**Table 3:** Molecular prevalence studies of *Anaplasma phagocytophilum* in questing ticks in Europe^*^.

**Country**	**Tick species**	**Year of tick collection**	**No. of ticks**	**Prevalence in %**	**Method**	**References**
Norway	*Ixodes spp*.	1998–1999	341	2.1^g^	PCR^a^	Jenkins et al., 2001
Norway	*Ixodes ricinus*		200	8.5		
			257	17.1		
		2006–2008^i^	145	3.4	qPCR^b^	Rosef et al., 2009
			235	0.4		
			348	14.9		
		2006	224	4.5	qPCR^b^	Radzijevskaja et al., 2008
		2011	87^adults^	4.6	qPCR^b^	Soleng and Kjelland, 2013
			133^nymphs^	0.8		
Sweden	*I. ricinus*	n.s.	151^nymphs^	6.6	PCR^a^	von Stedingk et al., 1997
		2007	1245^h^	11.5	qPCR^b^	Severinsson et al., 2010
Denmark	*I. ricinus*	1999–2000	106	23.6	PCR^a^	Skarphedinsson et al., 2007
Estonia	*I. ricinus*	2000	100	3	qPCR^a^	Mäkinen et al., 2003
		2006–2008	2474	1.7	qPCR^b^	Katargina et al., 2012
		2008–2010	112	2.7	nPCR^a^	Paulauskas et al., 2012
	*I. persulcatus*	2008–2010	31	6.5	nPCR^a^	Paulauskas et al., 2012
Latvia	*I. ricinus*	2008–2010	99	3.0	nPCR^a^	Paulauskas et al., 2012
	*I. persulcatus*	2008–2010	58	1.7	nPCR^a^	Paulauskas et al., 2012
Lithuania	*I. ricinus*	2006	140	3	qPCR^b^	Radzijevskaja et al., 2008
		2008–2010	277	2.9	nPCR^a^	Paulauskas et al., 2012
	*D. reticulatus*	2008–2010	87	8.0	nPCR^a^	Paulauskas et al., 2012
Russia	*I. ricinus*	1997–1998	295	13.6^g^	PCR^a^, RLB	Alekseev et al., 2001a
		2002	80	8.8	nPCR^b^	Masuzawa et al., 2008
		2006–2008	82	13.4	qPCR^b^	Katargina et al., 2012
	*I. persulcatus*	2002	84	16.7	qPCR^b^	Eremeeva et al., 2006
		2002	119	2.5	nPCR^b^	Masuzawa et al., 2008
Poland	*I. ricinus*	2000	424	19.2	PCR^a^	Stanczak et al., 2002
		1999	533	4.5	PCR^a^	Skotarczak et al., 2003
		2001	701	14	PCR^a^	Stanczak et al., 2004
		n.s.	694	13.1	PCR^a^	Tomasiewicz et al., 2004
		2002	174	4.6	PCR^a^	Rymaszewska, 2005
		2002	73	4.1	PCR^b^	Skotarczak et al., 2006
		2000–2004	1474	14.1	PCR^a^	Grzeszczuk and Stanczak, 2006
		2005	684	10.2	PCR^a^PCR^c^	Chmielewska-Badora et al., 2007
				2.8		
		2004–2006	1620^h^	4.9	PCR^a^	Wójcik-Fatla et al., 2009
		2007–2008	1123^h^	8.5	PCR^a^	Sytykiewicz et al., 2012
		n.s.	40	2.5	PCR^b^	Richter and Matuschka, 2012
Slovakia	*I. ricinus*	2002	60	8.3	PCR^a^	Derdáková et al., 2003
		2003–2004	271	4.4	PCR^a^	Smetanová et al., 2006
		2006	68	4.4^g^	PCR^a^	Špitalská et al., 2008
		n.s.	180	1.1	PCR^e^	Derdáková et al., 2011
			102	7.8		
		n.s.	80	8	qPCR^d^	Subramanian et al., 2012
Belarus	*I. ricinus*	2006–2008	187	4.2	qPCR^b^	Katargina et al., 2012
		2009	453	2.6	nPCR^f^	Reye et al., 2013
Ukraine	*I. ricinus*	2006	84	3.6	PCR^a^	Movila et al., 2009
Moldova	*I. ricinus*	2005	198	9	PCR^a^	Koèi et al., 2007
		2006	156	5.1	PCR^a^	Movila et al., 2009
Bulgaria	*I. ricinus*	2000	112^adults^	33.9	PCR^c^	Christová et al., 2001
			90^nymphs,^ ^h^	2.2		
Hungary	*I. ricinus*	2006–2008	1800^h^	0.4	nPCR^a^	Egyed et al., 2012
Serbia	*I. ricinus*	2001–2004	287	13.9	nPCR^b^	Tomanovic et al., 2010
		2007–2009	27	3.7^g^	PCR^a^	Tomanovic et al., 2013
	*D. reticulatus*	2007–2009	53	1.9^g^	PCR^a^	Tomanovic et al., 2013
	*Haemaphysalis concinna*	2007–2009	35	2.9^g^	PCR^a^	Tomanovic et al., 2013
Slovenia	*I. ricinus*	1996	93	3.2	PCR^a^	Petrovec et al., 1999
	*I. ricinus*	2005–2006	442^h^	0.6	PCR, nPCR^a^^,^^f^	Smrdel et al., 2010
UK (Scotland)	*I. ricinus*	1996–1997	210^h^	0.27–2.0	PCR^a^	Alberdi et al., 1998
		1996–1999	1476	3.0	PCR^a^	Walker et al., 2001
UK (Wales)	*I. ricinus*	n.s.	60	7.0	nPCR^a^	Guy et al., 1998
UK (England)	*I. ricinus*	n.s.	44^adults^	9	nPCR^a^	Ogden et al., 1998
			65^nymphs^	6		
	*I. ricinus*	n.s.	70^adults^	1.4	nPCR^a^	Ogden et al., 1998
			70^nymphs^	1.4		
	*I. ricinus*	2004–2005	4256^nymphs^	0.7	qPCR^b^	Bown et al., 2009
			263^females^	3.4		
			321^males^	2.5		
The Netherlands	*I. ricinus*	2000–2004	704	0.6	PCR^a^, RLB	Wielinga et al., 2006
Belgium	*I. ricinus*	2010	625	3.0	qPCR^a^^,^ ^j^	Lempereur et al., 2012
Luxembourg	*I. ricinus*	2007	1394	1.9	PCR^f^	Reye et al., 2010
France	*I. ricinus*	2003	4701^h^	15	PCR^a^	Halos et al., 2006
		2004	1065^nymphs^	0.4	PCR^a^	Ferquel et al., 2006
			171^adults^	1.2		
		2003	123^males^	4.3–9.4	nPCR^a^	Halos et al., 2010
			102^females^	2.2–10.7		
			3480^nymphs,^ ^h^	1.7–2.6		
		2006–2007	572	0.3	PCR^a^	Cotté et al., 2010
		2008	131	1.5	PCR^a^	Reis et al., 2011
Germany	*I. ricinus*	1999	492	1.6	PCR^a^	Fingerle et al., 1999
		2002	1963	2.6–3.1	nPCR^a^	Oehme et al., 2002
		2003	305	2.3	PCR^a^	Hildebrandt et al., 2002
		1999–2001	5424	1.0	nPCR^a^	Hartelt et al., 2004
		2003	127	3.9	PCR^a^, RLB	Pichon et al., 2006
		2006	2862	2.9	qPCR^b^	Silaghi et al., 2008
		2006–2007	1000	5.4	PCR^a^	Hildebrandt et al., 2010b
		2005	1646	3.2	qPCR^b^	Schicht et al., 2011
		2009–2010	5569	9.0^g^	qPCR^b^	Schorn et al., 2011
		n.s.	542	4.1	PCR^b^	Richter and Matuschka, 2012
		2009^i^	539	8.7		
			128	9.4	qPCR^b^	Silaghi et al., 2012b
			115	17.4		
		2011–2012	4064	5.3^g^	qPCR^b^	Overzier et al., 2013b
Austria	*I. ricinus*	2000–2001	235	5.1	PCR^a^	Sixl et al., 2003
		n.s.	880	8.7	qPCR^f^	Polin et al., 2004
Switzerland	*I. ricinus*	n.s.	100	2	qPCR^a^	Leutenegger et al., 1999
		1998	1667	1.3	qPCR^a^	Pusterla et al., 1999
		1998	417	1.4	nPCR^a^	Liz et al., 2000
		1999	6071^h^	1.2	qPCR^a^	Wicki et al., 2000
		2008	100^nymphs^	2	qPCR^b^	Burri et al., 2011
		2009–2010	1476	1.5	qPCR^b^	Lommano et al., 2012
Italy	*I. ricinus*	n.s.	86	24.4	PCR^a^	Cinco et al., 1997
		2002	1014	9.9	nPCR^a^	Mantelli et al., 2006
		2000–2001	1931	4.4	PCR^a^	Piccolin et al., 2006
		1998	55^h^	9	PCR	Lillini et al., 2006
		2010	232	8.2	qPCR^b^	Aureli et al., 2012
		2006–2008	193	1.5	qPCR^b^	Capelli et al., 2012
Spain	*I. ricinus*	2004	104^nymphs^	8.6	PCR^a^	Portillo et al., 2005
			54^adults^	3.7		
		2005–2006	168	10.7	nPCR^a^	Portillo et al., 2011
		2004	n.s.	20.5	PCR^a^	Ruiz-Fons et al., 2012
Portugal	*n.s*.	Archival collection	300	0.3	nPCR^f^	de Carvalho et al., 2008
	*I. ricinus*	2003–2004	142^h^	4.0	PCR^a^^,^^b^ PCR^b^	Santos et al., 2004
		n.s.	101	6.9		Richter and Matuschka, 2012
	*I. ventalloi*	2003–2004	93^h^	2.0	PCR^a^^,^^b^	Santos et al., 2004
Turkey European and Asian part)	*I. ricinus*	2008	241	2.7–17.5^*i*^	nPCR^a^^,^^b^	Sen et al., 2011

* *This table does not claim completeness. It does not include studies with 0% prevalence and studies with mixed results for questing and engorged tick*.

*nPCR, nested PCR; qPCR, real-time PCR; RLB, reverse line blot; n.s., not specified*.

a *16S rRNA as gene target*.

b *Msp2 as gene target*.

c *AnkA as gene target*.

d *ApaG as gene target*.

e *Msp4 as gene target*.

f *GroEL as gene target*.

g *Total prevalence not specified in the paper, prevalence was calculated by the authors of the present manuscript*.

h Study includes pools

i From different locations

j *Commercial kit*.

**Table 4:** Molecular prevalence studies of *Anaplasma phagocytophilum* in questing ticks in the USA^*^.

**State**	**Tick species**	**Year of tick collection**	**No. of ticks**	**Prevalence in %**	**Method**	**References**
New Hampshire	*Ixodes scapularis*	2007	509	0.2^e^	PCR	Walk et al., 2009
Rhode Island	*I. scapularis*	1996–1999	538	22.9	nPCR^a^	Massung et al., 2002
Connecticut	*I. scapularis*	1994	120	50.0	PCR^a^	Magnarelli et al., 1995
		1996–1997	1115	1.2–19.0^e^	PCR^a^	Levin et al., 1999
		1996–1999	375	13.3	nPCR^a^	Massung et al., 2002
New York	*I. scapularis*	2003–2004	25^females^	40.0	nPCR^c^	Moreno et al., 2006
			32^males^	50.0		
			62^nymphs^	27.0		
New Jersey	*I. scapularis*	2001	107	1.9	PCR^a^	Adelson et al., 2004
Pennsylvania	*I. scapularis*	2005	94	1.1	PCR^a^	Steiner et al., 2008
Wisconsin	*I. scapularis*	1998	636	3.8	PCR^a^	Shukla et al., 2003
		2006	100	14	nPCR^a^	Steiner et al., 2008
		2008	201	12.0	qPCR^b^	Lovrich et al., 2011
Indiana	*I. scapularis*	2003	68	11.8	nPCR^a^	Steiner et al., 2006
		2004	100	5	nPCR^a^	Steiner et al., 2008
Maine	*I. scapularis*	2003	100	16	nPCR^a^	Steiner et al., 2008
Maryland	*I. scapularis*	2003	348	0.3	PCR^a^	Swanson and Norris, 2007
Florida	*I. scapularis*	2004–2005	236	1.3	PCR^b^	Clark, 2012
	*Amblyomma americanum*	2004–2005	223	2.7	PCR^b^	Clark, 2012
Georgia	*I. scapularis*	2004–2005	808	20.0	nPCR^d^	Roellig and Fang, 2012
California	*Ixodes pacificus*	1995–1996	1112^adults,^ ^f^	0.8	nPCR^a^	Barlough et al., 1997a
			47^nymphs,^ ^f^	2.1		
		1997	84	1.2^e^	PCR^c^	Nicholson et al., 1999
		1996–1997	401^f^	2.0	nPCR^a^	Kramer et al., 1999
		1998	465^adults^	0	PCR^a^	Lane et al., 2001
			202^nymphs^	9.9		
		2000–2001	776	6.2	PCR^b^	Holden et al., 2003
		2002	234	3.4	nPCR^a^	Lane et al., 2004
		2000–2001	168	3.0	PCR^b^	Holden et al., 2006
		2005–2007	138	2.2^e^	qPCR^b^	Rejmanek et al., 2011
	*Dermacentor variabilis*	2000–2001	58	8.6	PCR^b^	Holden et al., 2003
	*D. occidentalis*	2000–2001	353	1.1	PCR^b^	Holden et al., 2003
		2003–2005; 2009–2010	513	0.2	nPCR^a^	Lane et al., 2010

* *This table does not claim completeness. It does not include studies with 0% prevalence and studies with mixed results for questing and engorged ticks*.

*nPCR, nested PCR; qPCR, real-time PCR; n.s., not specified*.

a *16S rRNA as gene target*.

b *Msp2 as gene target*.

c *GroESL as gene target*.

d *AnkA as gene target*.

e *Calculated by the authors of the present manuscript*.

f *Study includes pools*.

**Table 5:** Molecular prevalence studies of *Anaplasma phagocytophilum* in questing ticks in Asia^*^.

**Country**	**Tick species**	**Year of tick collection**	**No. of ticks**	**Prevalence in %**	**Method**	**References**
Russia	*Ixodes persulcatus*	2003–2004	125	2.4	nPCR^a^	Rar et al., 2005
		2002	8	12.5	PCR^a^	Shpynov et al., 2006
		2003–2010	3751	3.0	nPCR^a^	Rar et al., 2011
China	*I. persulcatus*	1997	372^d^	0.8^*^	nPCR^a^	Cao et al., 2000
		1999–2001	1345	4.6	nPCR^a^	Cao et al., 2003
		2005	100	4.0	nPCR^a^	Cao et al., 2006
	*Dermacentor silvarum*	2005	286	0.7	nPCR^a^	Cao et al., 2006
Japan	*I. persulcatus*	n.s.	325	6.2	PCR^b^	Murase et al., 2011
		2010–2011	134	21.6^f^	nPCR^a^	Ybañez et al., 2012
	*Haemaphysalis megaspinosa*	2008	48	12.5	nPCR^a^	Yoshimoto et al., 2010
	*H. douglasii*	2011	35	6.3^f^	nPCR^c^	Ybañez et al., 2013
	*I. persulcatus, I. ovatus*	n.s.	130	4.6^e^	nPCR^b^	Wuritu et al., 2009
Korea	*H. longicornis*	2004	241^d^	1.1	nPCR^a^	Chae et al., 2008
	*I. nipponensis*	2004	5^male^	20	nPCR^a^	Chae et al., 2008

* *This table does not claim completeness. It does not include studies with 0% prevalence and studies with mixed results for questing and engorged tick*.

*nPCR, nested PCR; n.s., not specified*.

a *16S rRNA gene as target*.

b *Msp2 gene as target*.

c *GroEL gene as target*.

d *Study includes pools*.

e *I. persulcatus and I. ovatus*.

f *Total prevalence not specified in the paper, prevalence was calculated by the authors of the present manuscript*.

Based on molecular detection in questing ticks, *A. phagocytophilum* seems to appear in all countries across Europe. In the US, the majority of studies have been performed in Eastern and Western (California) parts. From Northern US such data are lacking for several geographical regions, however serological evidence indicate exposure to *A. phagocytophilum* in large parts of the continent (Dugan et al., 2006; Bowman et al., 2009; Villeneuve et al., 2011). Two recent studies revealed the presence of *A. phagocytophilum* in questing ticks also in the Southern US (Florida and Georgia) (Clark, 2012; Roellig and Fang, 2012). Only few studies have been carried out in Asia, namely in Russia, China, Japan, and Korea (Table 5). It seems likely that other parts of Asia also belong to the endemic area of this pathogen.

Additionally to the ticks mentioned above, molecular detections have been reported from the following tick species (collected engorged from animals): *I. hexagonus*, *I. trianguliceps*, *I. spinipalpis*, *I. ochotonae*, and *D. nutalli* (Zeidner et al., 2000; Bown et al., 2003; Foley et al., 2011; Yaxue et al., 2011; Silaghi et al., 2012a). However, the vector competence of a lot of the tick species in which *A. phagocytophilum* has been detected as well as their contribution to the endemic cycle of *A. phagocytophilum* remain to be investigated.

The tick species *I. ricinus, I. persulcatus, I. scapularis*, and *I. pacificus* are found ubiquitously in their distribution range, have an open questing behavior and a broad host range, including many mammalian species (Sonenshine, 1993). These tick species may consequently also transmit the bacterium from animal reservoir hosts to humans. Aside from these aforementioned antropophilic and exophilic ticks, the involvement of nidicolous, and more host-specific endophilic ticks have been discussed in the context of so-called niche cycles, which may additionally keep the infection in nature. Examples for such proposed niche cycles involve cottontail rabbits (*Sylvilagus* spp.), *I. dentatus* and *I. scapularis* in the US (Goethert and Telford, 2003); field voles (*Microtus agrestis*), *I. trianguliceps* and *I. ricinus* in the UK (Bown et al., 2003); and hedgehogs (*Erinaceus europaeus*), *I. hexagonus* and *I. ricinus* in Europe (Silaghi et al., 2012a). The mentioned animals harbor two to three developmental stages of both endophilic and exophilic tick species and can thus transmit the agent from the animal host to humans via the anthropophilic tick species. Considering the large number of host specific and/or nidicolous ticks all around the world, it is likely that more potential niche cycles will be uncovered in the future (Foley et al., 2011).

Due to the comparatively low prevalence of *A. phagocytophilum* in *I. pacificus* in the Western US, *I. spinipalpis* has been suggested as a bridging vector for HGA (Zeidner et al., 2000). This nidiculous tick species infests, among others, Mexican woodrats (*Neotoma mexicana*) (in which *A. phagocytophilum* DNA has also been detected) and also occasionally bites humans and may thus transmit the agent from zooendemic cycles to humans.

Infection rates reported in many studies are higher in adult ticks than in nymphs. Due to the transstadial transmission, but lack of transovarial transmission, larvae are considered free of *A. phagocytophilum*. Adult ticks have had an additional blood meal in comparison to nymphs, and thus twice the chance of acquiring the infection. Variations in prevalence in questing ticks have also been observed with regard to the year of collection and in-between study areas and different geographic locations (Levin et al., 1999; Wicki et al., 2000; Hildebrandt et al., 2002; Cao et al., 2003; Holman et al., 2004; Ohashi et al., 2005; Grzeszczuk and Stanczak, 2006; Wielinga et al., 2006; Silaghi et al., 2008, 2012b; Schorn et al., 2011; Overzier et al., 2013b).

When looking at these variations, it has to be taken into account, that variations can be due to local variations, such as habitat structure or host availability, variation in methodology and sampling approach. Most studies shown in Tables 3–5 are single studies providing a spot prevalence, while studies including longitudinal data are scarce.

Variations in the prevalence of *A. phagocytophilum* in ticks may be attributed to several factors, such as the susceptibility of individual tick species, the susceptibility of certain tick populations, and the vector competence of tick species; the transmissibility of the *A. phagocytophilum* variant involved, the susceptibility of different host species, the susceptibility of individual hosts or host populations and the reservoir competence of the host. Especially the availability of different reservoir hosts and the adaptation strategy of *A. phagocytophilum* seem to be crucial factors in this variability. The availability of reservoir hosts depends on factors such as landscape structure and fragmentation (Medlock et al., 2013). In addition, effects exerted by changes in climate, demography, and agriculture may influence the tick distribution and density and their hosts.

## Hosts and reservoirs

Viable *A. phagocytophilum* organisms have been isolated from several hosts, such as cattle, sheep, goat, dog, horse, human, red deer (*Cervus elaphus*), roe deer (*Caperolus capreolus*), and white-tailed deer (WTD) (*Odocoileus virginianus*) (Foggie, 1951; Goodman et al., 1996; Munderloh et al., 1996; Woldehiwet et al., 2002; Massung et al., 2007; Stuen et al., 2010; Silaghi et al., 2011c). However, several prerequisites have to be fulfilled for a reservoir to be competent for a transstadially transmitted pathogen. A reservoir host must be fed on by an infected vector tick; it must take up a critical number of the infectious agent; it must allow the pathogen to multiply and survive for a period and it must allow the pathogen to find its way into other feeding ticks (Kahl et al., 2002). Several mammals may serve as hosts and reservoirs.

### Wild ruminants

In Europe, Asia, and America, *A. phagocytophilum* has been detected in local wild ruminant species (Tables 6–8). Wild ruminants such as WTD and roe deer are among the major feeding hosts for ticks in the Eastern US and Europe, respectively, and thus considered to contribute to a rapid increase in the population of ticks (Spielman et al., 1985; Vázquez et al., 2011; Medlock et al., 2013). WTD is considered one of the major reservoir hosts for an apathogenic variant (Ap-V1) of *A. phagocytophilum* in the Eastern US (Massung et al., 2005). Several genetic variants of *A. phagocytophilum* have been found in roe deer in Europe and there seem to be both potentially pathogenic and apathogenic variants occurring in roe deer (Silaghi et al., 2011b; Overzier et al., 2013a). A high roe deer density is associated with a high tick density (Jensen et al., 2000; Carpi et al., 2008; Rizzoli et al., 2009) and both presence and high density of roe deer seems to have a positive effect on the *A. phagocytophilum* prevalence (Rosef et al., 2009). Similarly, the density of WTD influences the density of *I. scapularis* ticks in the north-eastern US (Rand et al., 2003). For example, the elimination of WTD from certain areas lead to a drastic reduction of the occurrence of *I. scapularis* (Wilson et al., 1988). In a later study, however, there was no direct effect of a deer culling program on the occurrence of *I. scapularis* developmental stages (Jordan et al., 2007).

**Table 6:** DNA-Detection of *Anaplasma phagocytophilum* in blood/spleen in vertebrate hosts in the Americas^*^.

**Group of animals**	**Animal species**	**Country**	**No. of investigated**	**Prevalence in %**	**Method**	**References**
Wild ruminants	White-tailed deer (*Odocoileus virginianus*)	USA	458	16.0	PCR^a^^,^^b^	Dugan et al., 2006
		USA (Wisconsin)	181	15	PCR^a^	Belongia et al., 1997
		USA (Minnesota)	266	46.6	PCR^b^	Johnson et al., 2011
		USA (Connecticut)	63	37.0	PCR^b^	Magnarelli et al., 1999
		USA (Pennsylvania)	38	28.9	nPCR^a^	Massung et al., 2005
		USA (Wisconsin)	18	5.6	PCR^b^	Michalski et al., 2006
			40	22.5		
		USA (Mississippi)	32	3.1	PCR^b^	Castellaw et al., 2011
	Black-tailed deer (*Odocoileus hemonius columbianus*)	USA (California)	15	26.7^d^	nPCR^a^	Foley et al., 1998
	Mule deer (*O. h. hemonius*)	USA (California)	6	83.3^d^	nPCR^a^	Foley et al., 1998
	Elk (*Cervus elaphus nannodes*)	USA (California)	29	31.0	nPCR^a^	Foley et al., 1998
Small mammals (rodents)	White-footed mouse (*Peromyscus leucopus*)	USA (Minnesota)	158	11.4	nPCR^a^	Walls et al., 1997
			98–150	20.0–46.8	PCR^b^	Johnson et al., 2011
		USA (Connecticut)	47	36.2	nPCR^a^	Stafford et al., 1999
			135	14.1	PCR^b^	Levin et al., 2002
	Meadow jumping mouse (*Zapus hudsonius*)	USA (Minnesota)	18	50.0	PCR^b^	Johnson et al., 2011
	Cotton mouse (*P. gossypinus*)	USA (Florida)	41	4.9	PCR^b^	Clark, 2012
	Deer mouse (*P. maniculatus*)	USA (Colorado)	63	20.6	PCR^a^	Zeidner et al., 2000
			55^d^	9.2^d^	PCR^b^	DeNatale et al., 2002
	Brush mouse (*P. boylii*)	USA (California)	n.s.	4.0	qPCR^b^	Foley et al., 2008b
	Pinyon mouse (*P. truei*)	USA (California)	5^e^	20.0	PCR^c^	Nicholson et al., 1999
	Western harvest mouse (*Rheithrodontomys megalotis*)	USA (California)	n.s.	6.3	qPCR^b^	Foley et al., 2008b
	Red-backed vole (*Clethrionomys gapperi*)	USA (Minnesota)	6	17.0	nPCR^a^	Walls et al., 1997
			73	15.1	PCR^b^	Johnson et al., 2011
	Meadow vole (*Microtus pennsylvanicus*)	USA (Minnesota)	14	14.3	PCR^b^	Johnson et al., 2011
	Prairie vole (*Microtus ochrogaster*)	USA (Colorado)	15	6.6	PCR^a^	Zeidner et al., 2000
	Eastern chipmunk (*Tamias striatus*)	USA (Minnesota)	23	4.3	nPCR^a^	Walls et al., 1997
		USA (Rhode Island)	19	57.9	nPCR^a^	Massung et al., 2002
	Chipmunk	USA (Minnesota)	43	88.4	PCR^b^	Johnson et al., 2011
	Least chipmunk (*T. minimus*)	USA (Colorado)	5	40.0	PCR^b^	DeNatale et al., 2002
	Redwood chipmunk (*T. ochrogenys*)	USA (California)	60	6.6	qPCR^b^	Nieto and Foley, 2008
			n.s.	6.9	qPCR^b^	Foley et al., 2008b
			141	10.6	qPCR^b^	Foley and Nieto, 2011
	Sonoma chipmunk (*T. sonomae*)	USA (California)	5	40	qPCR^b^	Nieto and Foley, 2008
			n.s.	50.0	qPCR^b^	Foley et al., 2008b
	Chipmunk	USA (California)	81	8.9	qPCR^b^	Foley et al., 2011
	*Tamias* sp.	USA (California)	50	16.7^d^	qPCR^b^	Rejmanek et al., 2011
	Golden-mantled ground squirrel (*Spermophilus lateralis*)	USA (Colorado)	8	13	PCR^b^	DeNatale et al., 2002
	Eastern gray squirrel (*Sciurus carolinensis*)	USA (California)	27	11.1	qPCR^b^	Nieto and Foley, 2008
			n.s.	18.8	qPCR^b^	Foley et al., 2008b
			9	11.1^d^	qPCR^b^	Nieto et al., 2010
	Western gray squirrel (*S. griseus*)	USA (California)	41	12.1	qPCR^b^	Nieto and Foley, 2008
			n.s.	15.8	qPCR^b^	Foley et al., 2008b
			37	10.8^d^	qPCR^b^	Nieto et al., 2010
			6^e^	n.a.	qPCR^b^	Foley et al., 2008a
	Douglas squirrel (*Tamiasciurus douglasii*)	USA (California)	2^e^	n.a.	qPCR^b^	Foley et al., 2008a
	Northern flying squirrel (*Glaucomys sabrinus*)	USA (California)	20	5	qPCR^b^	Nieto and Foley, 2008
			n.s.	16.7	qPCR^b^	Foley et al., 2008b
			24	4.2^d^	qPCR^b^	Foley et al., 2007
			4	25.0^d^	qPCR^b^	Rejmanek et al., 2011
	Cotton rat (*Sigmodon hispidus*)	USA (Florida)	31	45.2	PCR^b^	Clark, 2012
	Mexican wood rat (*Neatoma mexicana*)	USA (Colorado)	36	38.8	PCR^a^	Zeidner et al., 2000
			30^d^	15^d^	PCR^b^	DeNatale et al., 2002
	Dusky-footed woodrat (*Neatoma fuscipes*)	USA (California)	25^e^	68	PCR^c^	Nicholson et al., 1999
			35^e^^,^ ^f^	68.6	PCR^c^	Castro et al., 2001
			134	71	qPCR^b^	Drazenovich et al., 2006
			n.s.	4.3	qPCR^b^	Foley et al., 2008b
			42	11.8	qPCR^b^	Foley et al., 2011
			53	9.4^d^	qPCR^b^	Rejmanek et al., 2011
	Big free-tailed bat (*Nyctinomops macrotis*)	USA (California)	n.s.	1.8	qPCR^b^	Foley et al., 2008b
Small mammals (insectivores)	Short-tailed shrew (*Blarina* spp.)	USA (Minnesota)	29	17.2	PCR	Johnson et al., 2011
Reptiles and Snakes	Northern alligator lizard (*Elgaria coeruleus*)	USA (California)	3	33.3	qPCR^b^	Nieto et al., 2009
	Sage-brush lizard (*Sceloporus graciosus*)	USA (California)	4	25.0	qPCR^b^	Nieto et al., 2009
	Western fence lizard (*S. occidentalis*)	USA (California)	77	9.1	qPCR^b^	Nieto et al., 2009
	Pacific gopher snake (*Pituophis catenifer*)	USA (California)	5	20.0	qPCR^b^	Nieto et al., 2009
	Common garter snake (*Thamnophis sirtalis*)	USA (California)	1	100	qPCR^b^	Nieto et al., 2009
Other	Cottontail rabbit (*S. floridanus*)	USA (Massachusetts)	203	27	nPCR^a^	Goethert and Telford, 2003
	American black bear	USA (California)	80	4	qPCR^b^	Drazenovich et al., 2006
	Gray Fox (*Urocyon cinereoargenteus*)	USA (California)	70^f^	9	qPCR^b^	Gabriel et al., 2009
	Raccoon (*Procyon lotor*)	USA (Connecticut)	57	24.6	PCR^b^	Levin et al., 2002
Domestic animals	Cat (stray)	USA (Connecticut)	6	33.3	PCR^b^	Levin et al., 2002
	Dog	USA (Minnesota)	222	3	PCR^a^	Beall et al., 2008
			51^g^	37		
		USA (California)	97	7	qPCR^b^	Drazenovich et al., 2006
			184	7.6	qPCR^b^	Henn et al., 2007
		Brazil	253	7.1	qPCR^b^	Santos et al., 2011
	Horse	Guatemala	74	13	nPCR^a^	Teglas et al., 2005
	Cattle	Guatemala	48	51	nPCR^a^	Teglas et al., 2005

* *This table does not claim completeness. It does not include studies with 0% prevalence and case reports*.

*nPCR, nested PCR; qPCR, real-time PCR; n.s., not specified*.

a *16S rRNA as gene target*.

b *Msp2 as gene target*.

c *GroEL as gene target*.

d *Total prevalence/number not specified in the paper, prevalence/number was calculated by the authors of the present manuscript*.

e *Seropositive for Anaplasma phagocytophilum antibodies*.

f *Includes recaptures*.

g *Partially with symptoms*.

**Table 7:** Detection of DNA of *Anaplasma phagocytophilum* in blood or tissue (majority spleen) of vertebrate hosts in Europe^*^.

**Group of animals**	**Animal species**	**Country**	**No. of investigated**	**Prevalence in %**	**Method**	**References**
Wild ruminants	Roe deer (*Capreolus capreolus*)	Denmark	237	42.6	qPCR^b^	Skarphedinsson et al., 2005
		UK	112	38.0	PCR^d^, SB	Alberdi et al., 2000
			279	47.3	qPCR^b^	Bown et al., 2009
			5	20.0	qPCR^b^	Robinson et al., 2009
		Poland	166	9.6	PCR^a^^,^^c^	Michalik et al., 2009
			31	38.7	nPCR^a^	Hapunik et al., 2011
		Slovakia	2	50.0	PCR^a^	Smetanová et al., 2006
			30	50.0	PCR^a^	Stefanidesová et al., 2008
		Czech Republic	40	12.5	qPCR^a^	Hulínská et al., 2004
			10	30.0	nPCR^a^	Zeman and Pecha, 2008
		Germany	31	94.0	nPCR^a^	Scharf et al., 2011
			95	98.9	qPCR^b^	Overzier et al., 2013a
		Austria	121	43.0	qPCR^d^	Polin et al., 2004
			19	52.6	qPCR^b^	Silaghi et al., 2011b
		Switzerland	103	18.4	nPCR^a^	Liz et al., 2002
		Italy	96	19.8	PCR^a^	Beninati et al., 2006
			8	50.0	PCR^a^^,^^e^	Torina et al., 2008b
		Spain	29	38.0	nPCR^a^	Oporto et al., 2003
			17	18.0	PCR^e^	de la Fuente et al., 2008
	Red deer (*Cervus elaphus*)	Norway	8	87.5^g^	nPCR^a^	Stuen et al., 2013
		UK	5	80.0	qPCR^b^	Robinson et al., 2009
		Poland	88	10.2	PCR^a^^,^^c^	Michalik et al., 2009
			106	50.9	nPCR^a^	Hapunik et al., 2011
		Czech Republic	15	13.3	qPCR^a^	Hulínská et al., 2004
			21	86.0	nPCR^a^	Zeman and Pecha, 2008
		Slovakia	3	33.3^g^	PCR^a^	Smetanová et al., 2006
			49	53.1	PCR^a^	Stefanidesová et al., 2008
		Austria	7	28.6	qPCR^d^	Polin et al., 2004
			12	66.7	qPCR^b^	Silaghi et al., 2011b
		Spain	21	23.8^g^	nPCR^a^	Portillo et al., 2011
	Iberian red deer (*C. e. hispanicus*)	Spain	6	100	PCR^e^	Naranjo et al., 2006
	Fallow deer (*Dama dama*)	UK	58	21.0	qPCR^b^	Robinson et al., 2009
		Poland	44	20.5	PCR^a^^,^^c^	Michalik et al., 2009
			130	1.5	nPCR^a^	Hapunik et al., 2011
			50	14.0^g^	PCR^a^	Adaszek et al., 2012
		Czech Republic	15	13.3	PCR^a^	Hulínská et al., 2004
			2	50.0	nPCR^a^	Zeman and Pecha, 2008
		Italy	72	15.3	PCR^a^	Veronesi et al., 2011
			29	72.4	nPCR^a^	Ebani et al., 2011
	Sika deer (*Cervus nippon*)	UK	12	50.0	qPCR^b^	Robinson et al., 2009
		Poland	32	34.4	nPCR^a^	Hapunik et al., 2011
		Czech Republic	5	40.0	nPCR^a^	Zeman and Pecha, 2008
	Chamois (*Rupicapra rupicapra*)	Austria	23	26.1	qPCR^b^	Silaghi et al., 2011b
	Alpine ibex (*Capra ibex*)	Austria	18	16.7	qPCR^b^	Silaghi et al., 2011b
	Mouflon (*Ovis musimon*)	Czech Republic	28	4.0	nPCR^a^	Zeman and Pecha, 2008
			15	13.3	PCR^a^	Hulínská et al., 2004
		Slovakia	2	50.0	PCR^a^	Stefanidesová et al., 2008
		Austria	6	50.0	qPCR^b^	Silaghi et al., 2011b
	European bison (*Bison bonasus*)	Poland	26	58.0	nPCR^a^	Scharf et al., 2011
			5	57.7^g^	nPCR^a^	Matsumoto et al., 2009
Small mammals (rodents)	Yellow necked-mouse (*Apodemus flavicollis*)	Czech Republic	40	15.0	qPCR^a^	Hulínská et al., 2004
		Slovakia	38	5.3^g^	PCR^a^	Smetanová et al., 2006
		Germany	218	0.5	nPCR^a^	Hartelt et al., 2008
		Switzerland	69	2.9	nPCR^a^	Liz et al., 2000
	Wood mouse (*A. sylvaticus*)	UK	902^j^	0.8	nPCR^a^	Bown et al., 2003
		Switzerland	48	4.2	nPCR^a^	Liz et al., 2000
		France	18	11.1^g^	PCR^a^	Matsumoto et al., 2007
		Spain	162	0.6	PCR^b^, RLB	Barandika et al., 2007
	Black-striped field mouse (*A. agrarius*)	Bulgaria	9	33.3	PCR^c^	Christová and Gladnishka, 2005
	Bank vole (*Myodes glareolus*)	UK	527	5.0	nPCR^a^	Bown et al., 2003
		Czech Republic	15	13.3	qPCR^a^	Hulínská et al., 2004
		Switzerland	78	19.2	nPCR^a^	Liz et al., 2000
		Germany	149	13.4	nPCR^a^	Hartelt et al., 2008
			36	5.5	qPCR^b^	Silaghi et al., 2012b
	Common vole (*Microtus arvalis*)	Germany	97	6.2	nPCR^a^	Hartelt et al., 2008
	Field vole (*Mi. agrestis*)	UK	163	6.7	nPCR^a^	Bown et al., 2006
			2402^j^	6.7	qPCR^b^	Bown et al., 2008
			1503^j^	6.3	qPCR^b^	Bown et al., 2009
	Root vole (*Mi. oeconomus*)	Poland	30	6.7^g^	nPCR^a^	Grzeszczuk et al., 2006
	Black rat (*Rattus rattus*)	Bulgaria	136	4.4	PCR^c^	Christová and Gladnishka, 2005
	Porcupine (Hystricidae)	Italy	1	100	PCR^a^	Torina et al., 2008a
Small mammals (insectivores)	Common shrew (*Sorex araneus*)	UK	76	1.3	PCR^a^	Bray et al., 2007
			647^j^	18.7	qPCR^b^	Bown et al., 2011
		Switzerland	5	20.0^g^	nPCR^a^	Liz et al., 2000
	European hedgehog (*Erinaceus europaeus*)	Germany	31	25.8	nPCR^a^	Skuballa et al., 2010
			48	85.4^g^	qPCR^b^	Silaghi et al., 2012a
	Greater white-toothed shrew (*Crocidura russula*)	Spain	6	16.7	PCR^b^, RLB	Barandika et al., 2007
Birds	Blackbird (*Turdus merula*)	Spain	3	100	PCR^e^	de la Fuente et al., 2005b
	Chaffinch (*Fringilla coelobs*)	Spain	1	100	PCR^e^	de la Fuente et al., 2005b
	House sparrow (*Passer domesticus*)	Spain	18	6.0	PCR^e^	de la Fuente et al., 2005b
	Spanish Sparrow (*Passer hispaniolensis*)	Spain	3	33.0	PCR^e^	de la Fuente et al., 2005b
	Rock bunting (*Emberiza cia*)	Spain	1	100	PCR^e^	de la Fuente et al., 2005b
	Woodchat shrike (*Lanius senator*)	Spain	1	100	PCR^e^	de la Fuente et al., 2005b
	Magpie (*Pica pica*)	Spain	1	100	PCR^e^	de la Fuente et al., 2005b
	Long-tailed tit (*Aegithalos caudatus*)	Spain	1	100	PCR^e^	de la Fuente et al., 2005b
Other	European Brown bear (*Ursus arctos*)	Slovakia	74	24.3	PCR^a^	Vichová et al., 2010
	Red fox (*Vulpes vulpes*)	Poland	111	2.7	nPCR^a^	Karbowiak et al., 2009
		Czech Republic	25	4.0	PCR^a^	Hulínská et al., 2004
		Italy	150	16.6	nPCR^a^	Ebani et al., 2011
	Wild boar (*Sus scrofa*)	Poland	325	12	nPCR^a^	Michalik et al., 2012
		Slovakia	18	5.5^g^	PCR^a^	Smetanová et al., 2006
		Czech Republic	69	4.4	PCR^a^	Hulínská et al., 2004
		Slovenia	113	2.7^g^	PCR^a^	Galindo et al., 2012
			160	6.3	qPCR^f^	Zele et al., 2012
	Hare (*Leparus europaeus*)	Czech Republic	8	12.5	PCR^a^	Hulínská et al., 2004
Domestic animals	Cat	Germany	306	0.3^g^	qPCR^b^	Hamel et al., 2012a
		Germany	265	0.4	qPCR^b^	Morgenthal et al., 2012
	Dog	UK	120^k^	0.8^g^	PCR^a^	Shaw et al., 2005
		Poland	408	0.5	PCR^c^	Zygner et al., 2009
			242^k^	5.4	PCR^b^	Rymaszewska and Adamska, 2011
		Czech Republic	296^k^	3.4	nPCR^a^	Kybicová et al., 2009
		Germany	111	6.3	nPCR^a^	Jensen et al., 2007
			522^k^	5.7	qPCR^b^	Kohn et al., 2011
		Italy	46	2.8–21.7^i^	PCR^a^^,^^e^	Torina et al., 2008a
		Italy (Sardinia)	50^k^	7.5^g^	nPCR^d^	Alberti et al., 2005a
		Hungary/Romania	216	1.9	qPCR^b^	Hamel et al., 2012b
	Horse	Czech Republic	40	5	PCR^a^	Hulínská et al., 2004
		Netherlands	61^k^	9.8^g^	PCR^a^, RLB	Butler et al., 2008)
		Italy	135^k^	8.1^g^	nPCR^a^	Passamonti et al., 2010
			5^k^	80.0^g^	PCR	Lillini et al., 2006
			134	0–4.7^i^	PCR^a^^,^^e^	Torina et al., 2008a
			300	6.7^g^	PCR^a^	Laus et al., 2013
			42	4.7	PCR^a^^,^ ^e^	Giudice et al., 2012
		Italy (Sardinia)	20^k^	15.0^g^	nPCR^d^	Alberti et al., 2005a
	Donkey	Italy	76	4	PCR^a^^,^ ^e^	Torina et al., 2008b
		Spain	3	100	PCR^e^	Naranjo et al., 2006
	Cattle	Czech Republic	55	5.5	PCR^a^	Hulínská et al., 2004
		France	20^j^	20.0^g^	PCR^a^^,^ ^d^^,^ ^e^	Laloy et al., 2009
		Switzerland	27^k^ 16^k^	4.0 13.0	qPCR^a^	Hofmann-Lehmann et al., 2004
		Italy	78	17	PCR^a^^,^ ^e^	Torina et al., 2008b
			374	0–2.9^i^	PCR^a^^,^ ^e^	Torina et al., 2008a
		Spain	107	19	PCR^e^	de la Fuente et al., 2005b
			157	13	PCR^e^	Naranjo et al., 2006
	Sheep	Norway	32	37.5^g^	nPCR^a^^,^ ^e^	Stuen et al., 2013
		Denmark	43	11.6^g^	PCR^a^	Kiilerich et al., 2009
		Germany	255	4	nPCR^a^	Scharf et al., 2011
		Italy	200	11.5	PCR^a^	Torina et al., 2010
			286	0–3.8^i^	PCR^a^^,^ ^e^	Torina et al., 2008a
			90	3	PCR^a^	Torina et al., 2008b
	Sheep, goats	Slovakia, Czech Republic	323	2.8^h^	PCR^e^	Derdáková et al., 2011
	Goats	Switzerland	72	5.6^g^	qPCR^b^	Silaghi et al., 2011e
		Italy	134	0–3.5^i^	PCR^a^^,^ ^e^	Torina et al., 2008a

* *This table does not claim completeness. It does not include studies with 0% prevalence and case reports*.

*nPCR, nested PCR; qPCR, real-time PCR; RLB, reverse line blot, SB, Southern Blot*.

a *16S rRNA as gene target*.

b *Msp2 as gene target*.

c *AnkA as gene target*.

d *GroEL as gene target*.

e *Msp4 as gene target*.

f *Commercial kit*.

g *Total prevalence not specified in the paper, prevalence was calculated by the authors of the present manuscript*.

h *Sheep only*.

i *Range represents confidence interval*.

j *Individuals sampled several times*.

k *Partially with symptoms*.

**Table 8:** Detection of DNA of *Anaplasma phagocytophilum* in spleen/blood of vertebrate hosts in Asia and Africa^*^.

**Group of animals**	**Animal species**	**Country**	**No. of investigated**	**Prevalence in %**	**Method**	**References**
**ASIA**						
Wild ruminants	Sika deer (*Cervus nippon*)	Japan	22	46.0	nPCR^a^	Jilintai et al., 2009
			126	19.0	nPCR^a^	Kawahara et al., 2006
			32	15.6	nPCR^a^	Masuzawa et al., 2011
	Korean water deer (*Hydropotes inermis argyropus*)	Korea	66	63.6	nPCR^a^	Kang et al., 2011
	Wood mouse (*Apodemus sylvaticus*)	China	20	10.0	nPCR^a^	Zhan et al., 2008
			21	9.5	nPCR^a^	Zhan et al., 2009a
	Black-striped field mouse (*Apodemus agrarius*)	China	24	20.8	nPCR^a^	Cao et al., 2006
			142	9.9	nPCR^a^	Zhan et al., 2009a
			78	12.8	qPCR^b^	Zhan et al., 2010
			12	16.7	nPCR^a^	Yang et al., 2013
		Korea	358	5.6	nPCR^a^	Chae et al., 2008
			373	23.6^d^	nPCR^a^	Kim et al., 2006
	Korean field mouse (*Apodemus peninsulae*)	Russia	359	0.6^d^	nPCR^a^	Rar et al., 2011
		China	43	7.0	nPCR^a^	Cao et al., 2006
			74	5.4	nPCR^a^	Zhan et al., 2009a
			4	25.0	qPCR^b^	Zhan et al., 2010
	Bank vole (*M. glareolus*)	Russia	61^d^	6.6^d^	nPCR^a^	Rar et al., 2011
	Red-backed vole (*Myodes rutilus*)	Russia	189^d^	14.8^d^	nPCR^a^	Rar et al., 2011
	Red gray-backed vole (*Myodes rufocanus*)	Russia	776^d^	5.2^d^	nPCR^a^	Rar et al., 2011
		China	65	4.6	nPCR^a^	Zhan et al., 2009a
	East-European field vole *(Microtus rossiaemeridionalis)*	Russia	38^e^	2.6^d^	nPCR^a^	Rar et al., 2011
	Brown house rat (*Rattus norvegicus*)	China	9	55.5	qPCR^b^	Zhan et al., 2010
			9	33.3	nPCR^a^	Zhan et al., 2008
	Chinese white bellied rat (*Niviventer confucianus*)	China	48	12.5	nPCR^a^	Zhan et al., 2008
			115	5.2	nPCR^a^	Zhan et al., 2009a
	White-bellied giant rat (*Niviventer coxingi*)	China	4	25.0	nPCR^a^	Zhan et al., 2008
			4	25.0	nPCR^a^	Zhan et al., 2009a
	Lesser rice field rat (*Rattus losea*)	China	2	50.0	nPCR^a^	Zhan et al., 2008
			32	3.1	nPCR^a^	Zhan et al., 2009a
	Brown rat (*R. norvegicus*)	China	47	8.5	nPCR^a^	Zhan et al., 2009a
	Siberian chipmunk (*Tamias sibiricus*)	Russia	24	25.0^d^	nPCR^a^	Rar et al., 2011
		China	3	33.3	nPCR^a^	Cao et al., 2006
			18	5.6	nPCR^a^	Zhan et al., 2009a
	Great long-tailed hamster (*Tscherskia triton*)	China	65	9.2	qPCR^b^	Zhan et al., 2010
	*Cricetulus* sp.	China	39	5.1	nPCR^a^	Zhan et al., 2009a
	Gray hamster (*Cricetulus migratorius*)	China	3	33.3	qPCR^b^	Zhan et al., 2010
Small mammals (insectivores)	White-toothed shrew (*Crocidura lasiura*)	Korea	33	63.6^d^	nPCR^a^	Kim et al., 2006
	Common shrew (*Sorex araneus*)	Russia	137^d^	4.4^d^	nPCR^a^	Rar et al., 2011
Other	Chinese hare (*Lepus sinensis*)	China	54	1.9	nPCR^a^	Zhan et al., 2009b
	Wild boar (*Sus scrofa*)	Japan	56	3.6	nPCR^a^	Masuzawa et al., 2011
Domestic animals	Dog	China	101	10.9	nPCR^a^	Zhang et al., 2012a
	Cattle	Japan	15	80.0	PCR^a^	Ooshiro et al., 2008
			78	1.0	nPCR^a^	Jilintai et al., 2009
			1251	3.4	PCR^b^	Murase et al., 2011
			50	2.0	nPCR^c^	Ybañez et al., 2013
		China	71	23.9	nPCR^a^	Zhang et al., 2012a
			201	23.4	nPCR^a^	Zhang et al., 2012a
	Yaks	China	158	32.3	nPCR^a^	Yang et al., 2013
	Cattle-yaks	China	20	35.0	nPCR^a^	Yang et al., 2013
	Sheep	China	70^f^	7.1	qPCR^b^	Zhan et al., 2010
			49	42.9	nPCR^a^	Yang et al., 2013
	Goat	China	35^f^	5.7	qPCR^b^	Zhan et al., 2010
			91	38.5	nPCR^a^	Yang et al., 2013
			90	48.9	nPCR^a^	Zhang et al., 2012b
			472	26.7	nPCR^a^	Zhang et al., 2012a
			262	6.1	nPCR^a^	Liu et al., 2012
**AFRICA**						
Domestic animals	Dog	Tunisia	228	0.9^d^	PCR^a^	M'Ghirbi et al., 2009
	Horse	Tunisia	60	13	nPCR^a^	M'Ghirbi et al., 2012

* *This table does not claim completeness. It does not include studies with 0% prevalence*.

*nPCR, nested PCR; qPCR, real-time PCR*.

a *16S rRNA gene as target*.

b *Msp2 gene as target*.

c *GroEL gene as target*.

d Total prevalence not specified in the paper, prevalence was calculated by the authors of the present manuscript

e *Microtus spp*.

f *Partially with symptoms*.

In the US, WTD has prevalence rates of *A. phagocytophilum* of up to 46.6% (Table 6), while detection of *A. phagocytophilum* in wild ruminants other than WTD are scarce so far. In Europe, roe deer show prevalence rates reaching up to 98.9% (Overzier et al., 2013a). Other deer species seem to contribute to the endemic cycles in Europe, and may also constitute efficient reservoir hosts, as the pathogen has been detected in red deer with up to 87% prevalence, in fallow deer (*Dama dama*) with up to 72%, and in sika deer (*Cervus nippon*) with up to 50% (Table 7). *A. phagocytophilum* has also been identified in deer species in Asia, namely sika deer and water deer (*Hydropotes inermis*) with prevalence rates of up to 46% and of 63.6%, respectively (Jilintai et al., 2009; Kang et al., 2011; Table 8). However, the studies that have been conducted in Asia on wild ruminants are too few as to draw any definite conclusion on the distribution of *A. phagocytophilum*.

### Small mammals

The second large group of animals that *A. phagocytophilum* is found in endemic countries are in small mammals such as rodents and insectivores. These animals also are major feeding hosts for ticks, especially for the developmental stages (Kiffner et al., 2011). DNA of *A. phagocytophilum* was found in different mouse, vole, other rodent and insectivore species in the US, Europe, and Asia (Tables 6–8).

#### Rodents

In Europe, yellow-necked mice (*Apodemus flavicollis*) were infected with ranges from <1 to 15%, wood mice (*Apodemus sylvaticus*) from <1 to 11% and bank voles (*Myodes glareolus*) from 5 to 19.2%. In mouse species, detection with higher prevalence rates represents only single studies, whereas detection in bank voles seemed higher and more consistent. This was also the case for other vole species in Europe (Table 6). In the UK, the field vole has been discussed as a potential small mammal reservoir (Bown et al., 2003). However, in several studies on rodents in Europe, no DNA of *A. phagocytophilum* has been detected or at such low prevalence rates, that a reservoir role of this group of animals in Europe remains unclear (Barandika et al., 2007; Silaghi et al., 2012b; Table 6).

On the contrary, in the Eastern US, the white-footed mouse (*Peromyscus leucopus*) is considered one of the major reservoir hosts for the human pathogenic variant (Ap-ha) (Massung et al., 2003). *P. leucopus* is found as the predominant small mammal in forested habitats throughout the Eastern and Central US and it is one of the major hosts for the larval stages of *I. scapularis* (Sonenshine, 1993). The white-footed mouse has reservoir competence for the AP-ha variant, but reservoir competence could not be shown for the apathogenic Ap-V1 variant (Massung et al., 2003), as opposed to the aforementioned WTD as a major reservoir hosts for Ap-V1 (Massung et al., 2005). Different lengths of infections with the two strains have also been shown in an experimental WTD study: Ap-V1 from tick cells resulted in lasting parasitemia, whereas infection with Ap-ha was short-lived (Reichard et al., 2009). By contrast, both Ap-V1 and Ap-ha were infectious for goats and goats are reservoir competent to Ap-V1 (Massung et al., 2006).

Ap-V1 was isolated from goats and *I. scapularis* and propagated in the ISE6 tick cell line, but it could not be cultivated in the human HL-60 cell line. This stands in contrast to *A. phagocytophilum* strains which have been isolated from human cases in the US, which readily grow in HL-60 cell lines (Horowitz et al., 1998; Massung et al., 2007), suggesting differing host specificity for these two strain types.

Apart from the white-footed mouse, *A. phagocytophilum* DNA has been detected in several rodent species such as voles and chipmunks in the Eastern US, cotton mice and cotton rats in Florida and several mouse-, chipmunk-, and squirrel-species as well as the dusky-footed woodrat (*Neotoma fuscipes*) in the Western US (Table 7). Prevalence ranges from 1.8 to 88.4%. The gray squirrel (*Sciurus carolinensis*) has also been found to be reservoir competent (Levin et al., 2002) and the redwood chipmunk (*Tamias ochrogenys*) and sciurid rodents are discussed as important reservoir hosts for *A. phagocytophilum* in the Western US (Nieto et al., 2010; Foley and Nieto, 2011). Similarly to other small mammals that have been suggested to maintain niche cycles, the redwood chipmunk hosts both antropophilic (*I. pacificus*) and nidicolous (*I. angustus*) ticks (Foley and Nieto, 2011).

In Asia, comparatively high prevalence rates in small mammals also seem to indicate a reservoir function of this group of mammals (Table 8). For example, in China, wood mice showed prevalence rates up to 10.0% (Zhan et al., 2008), Korean field mice (*A. peninsulae*) up to 25% (Zhan et al., 2010) and black-striped field mice (*A. agrarius)* up to 20.8% (Cao et al., 2006). In Korea, prevalence rates in the black-striped field mouse was also up to 23.6% (Kim et al., 2006) and therefore, *A. agrarius* has been discussed as one of the major reservoir host in Asian countries. In the Asian part of Turkey, however, all captured rodents were serologically negative for *A. phagocytophilum* (Güner et al., 2005).

Additionally to mice, voles, chipmunks, and squirrels, DNA of *A. phagocytophilum* has also been detected in rats on all three continents, in hamsters (China) and in a porcupine (Italy) (Tables 6–8).

#### Insectivores

There are very few published studies on the role of insectivores in the life cycle of *A. phagocytophilum*. The common shrew (*Sorex araneus*) has been discussed as a reservoir host for *A. phagocytophilum* in the UK (Bown et al., 2011). In that study, prevalence reached 18.7%. Other insectivores which have been investigated in Europe were the greater white-toothed shrew (*Crocidura russula*) and the European hedgehog (Table 6). DNA of *A. phagocytophilum* has also been detected in short-tailed shrews (*Blarina brevicauda*) with 17.2% prevalence in the US and in Asia in white-toothed shrews with 63.6% prevalence (Tables 6, 8). Detection rates of *A. phagocytophilum* in insectivores were generally high, with average prevalence rates around 20%, reaching over 80%. However, the role of insectivores in the life cycle of *A. phagocytophilum* needs further investigation.

### Other animal species

Apart from wild ruminants, rodents and insectivores, there are several other vertebrate species in which DNA from *A. phagocytophilum* has been isolated. Whether these contribute to the endemic cycle of *A. phagocytophilum* is currently not clear. Amongst these animals are mammals such as wild boars, foxes, and bears, but also birds and reptiles (Tables 6–8). The prevalence rates in these animal species seem similar to the potential reservoir hosts discussed above, but studies have been very few so a final conclusion is not yet possible. In the US, raccoons (*Procyon lotor***)** have been found to be reservoir competent for *A. phagocytophilum* (Levin et al., 2002; Yabsley et al., 2008), while wild boar (*Sus scrofa*) has recently been discussed as a host for human pathogenic variants of *A. phagocytophilum* in Europe (Michalik et al., 2012).

The questions which remain open are whether many different animal species get infected only temporarily with potentially non-species specific strains of *A. phaogcytophilum* and constitute dead-end hosts such as human beings, whether they develop clinical signs of disease or if they contribute in any way to the endemic cycle.

### Domestic animals

Dogs in Europe were positive for DNA of *A. phagocytophilum* at about 1–6% prevalence, regardless whether they show symptoms of canine granulocytic anaplasmosis or not. By comparison, the prevalence rates in cats were much lower, with <0.5%. In horses, prevalence was higher ranging up to 80%, however, several of the studies investigated horses with symptoms of equine granulocytic anaplasmosis. Without any clinical signs, the prevalence in horses was less than 6.7% (Tables 6–8). Furthermore, several case reports and case series have been published on domestic animals in North America (e.g., Cockwill et al., 2009; Granick et al., 2009; Uehlinger et al., 2011), and serological studies have shown a wide evidence of dogs, cats, and horses being in contact with *A. phagocytophilum* in USA, Canada, and Asia (e.g., Magnarelli et al., 2001; Billeter et al., 2007; Bowman et al., 2009; Villeneuve et al., 2011; Bell et al., 2012; Ybañez et al., 2012). Additionally, serological and molecular evidence have been provided from North Africa (which also is an endemic area for *I. ricinus*) that horses and dogs become infected with *A. phagocytophilum* (M'Ghirbi et al., 2009, 2012). This important finding broadens the known geographic range of *A. phagocytophilum* to Africa as another continent.

The role of dogs as reservoir hosts has been discussed (Schorn et al., 2011). Furthermore, a report of granulocytic anaplasmosis has been described in another member of the canine family, a captive timber wolf (*Canis lupus*) (Leschnik et al., 2012). The question remains open whether dogs can contribute to the natural cycle of *A. phagocytophilum*: Is the infection persistent enough for subsequent ticks to become infected, and do dogs host enough nymphal stages of ticks to contribute to the spread? Animals which host mainly adult ticks cannot effectively contribute to the life cycle of *A. phagocytophilum*, as transovarial infection does not seem to occur.

### Domestic ruminants

Infection with *A. phagocytophilum* has also been detected in several domestic ruminant species such as sheep, goats, cattle, and yaks (Tables 6–8). In Europe, domestic ruminants have been found infected with DNA with rates of up to 20% (cattle), 37% (sheep), and 5.6% (goats) (Table 6). However, larger scale molecular studies on domestic ruminants in Northern America are lacking, but cases of granulocytic anaplasmosis have been described in llama (*Lama glama*) and alpaca (*Vicugna pacos*) in California and Massachusetts, respectively (Barlough et al., 1997a,b; Lascola et al., 2009). Furthermore, serological evidence has been provided for *A. phagocytophilum* antibodies in cattle in Connecticut (Magnarelli et al., 2002).

## Spread of infection

*A. phagocytophilum* may be spread between different geographic regions by both infected ticks and infected hosts. Expansion of existing endemic areas or to new geographic regions occurs when populations of competent vectors and reservoirs or the abundance of susceptible hosts increase both in total number and in geographic range.

Roe deer carry large number of ticks and moves over long distances (Vor et al., 2010) and may therefore add to the spread of the pathogen itself as well as by moving infected ticks to other areas (Overzier et al., 2013a). Factors contributing to a wider occurrence of suitable hosts such as WTD, white-footed mice, roe deer, field mice etc. may be landscape changes leading to an expansion in the distribution range as well as in the density of those hosts.

Landscape changes such as reforestation may also lead to an expansion of the anthropophilic ticks which are spread also when their primary feeding hosts expand (Sonenshine, 1993).

The increase and spread of *I. scapularis* in the Eastern US has lead to an increase in Lyme Borreliosis cases (Sonenshine, 1993) and may similarly contribute to the expansion of *A. phagocytophilum*. In Europe, the increasing geographic range of *I. ricinus* as well as the expansion to higher altitudes has recently been discussed by several authors (Materna et al., 2005; Jore et al., 2011; Jaenson et al., 2012; Medlock et al., 2013).

**Figure 1:**
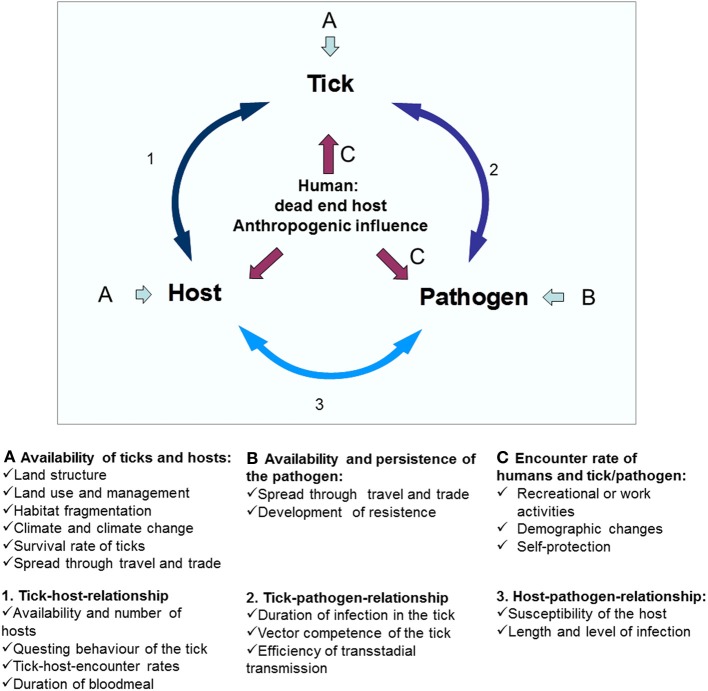
**Direct, indirect, and anthropogenic influences on the tick-host-pathogen relationship of *Anaplasma phagocytophilum***.

Domestic animals including pet animals such as the dog and farm animals such as sheep and cattle may be transported to other areas, in-between countries, even continents, and can thus also add to the spread of infection. Ticks may be spread by birds over long distances and with them *A. phagocytophilum*–infected ticks. Studies from Europe indicate that migrating birds may be important in the dispersal of *A. phagocytophilum* infected *I. ricinus* (Alekseev et al., 2001b; Bjöersdorff et al., 2001). However, *A. phagocytophium* DNA has sometimes been detected in ticks collected from birds at low prevalence, and it was questioned by some authors whether birds may really be involved in the spreading of the pathogen whereas other authors discussed their possible involvement (Daniels et al., 2002; Ogden et al., 2008; Franke et al., 2010; Hildebrandt et al., 2010a; Dubska et al., 2012; Palomar et al., 2012; Hornok et al., 2013; Kang et al., 2013). The involvement of birds and their ticks in the life cycle of *A. phagocytophilum* has also been tested in a transmission study in the US. For the two bird species [American robin (*Turdus migratorius*) and Gray catbird (*Dumetella carolinensis*)] involved, no significant role in the life cycle was found (Johnston et al., 2013). However, the establishment of ticks in a new habitat depends on the density of hosts in that area, the habitat structure, and the character of the local microclimate and its changes (Daniel, 1993). As an example of this complexity, Figure 1 shows a summary of several direct and indirect factors which are influencing the occurrence and the spread of *A. phagocytophilum* to humans.

## Geographic distribution and genetic variation

As already shown in Tables 3–8, *A. phagocytophilum* has a wide geographical distribution. However, there is a huge lack of knowledge on ecology, epidemiology and source attributions, vector biology and the clinical implication of different pathogenic strains, related to risk posed on animals and humans (Zhang et al., 2013). This intercepts with the development of effective prevention, control, and eradication strategies for *A. phagocytophilum*. As already mentioned, transovarial transmission does not seem to occur in tick species associated with infection of humans or animals and the dependence on reservoir animals for maintenance of infection in nature seems crucial (Ogden et al., 1998; Liz et al., 2002). Understanding the extent and mechanisms behind bacterial strain diversity, geographical distribution, and host-pathogen fitness on vector and animal level is increasingly important to give accurate estimates to veterinary and public health risks. Former and future developments in methodologies in molecular epidemiology and genetic fingerprinting like multi-locus sequence typing (MLST), pulse field gel electrophoresis (PFGE), high throughput genome sequencing, blood meal genetic analyses, and the study of microbiomes by for instance metagenomic analyses are powerful approaches to delineating bacterial population structures and the evolutionary processes that underlie these (Dumler et al., 2003; Bown et al., 2007; Dark et al., 2012).

*A. phagocytophilum* is currently viewed as a single bacterial species, seemingly capable of infecting a broad range of hosts based on *16S rRNA* gene analyses. The appearance of *16S rRNA* gene variants in ticks seems to be dependent on the habitat structure and therefore of the occurrence of specific potential reservoir hosts, which supports the theory of a host association of some variants (Overzier et al., 2013a,b). The situation appears to be even more complex and delicate in its partiality for certain hosts than previously foreseen, when high resolution methods are used to further delineate strains at host level. Strain variation with potential specific host tropism seems to be abundant in *A. phagocytophilum* and as such, this has to be taken into account when considering the spread of infection, and the contribution of wildlife such as wild ruminant species in infection cycles involving domestic animals and humans.

*A. phagocytophilum* is sometimes seen to circulate between hosts sharing similar ecological niches (Al-Khedery et al., 2012; Michalik et al., 2012). For example, phylogenetic investigations of the *groEL* gene have revealed a clustering of sequences into those from roe deer and those from others, as well as a clustering according to geographic origin (Alberti et al., 2005a,b; Silaghi et al., 2011c,d).

Investigations on several *A. phagocytophilum* strains from different hosts in California indicated that multiple unique strains of *A. phagocytophilum* with distinct host tropisms exist (Rejmanek et al., 2012). Furthermore, one study in the Western US showed no overlap in the endemic cycles found with variants from HGA cases and from the suggested wild-life reservoir, the dusky-footed wood rat (Foley et al., 2008a,b).

*A. phagocytophilum 16S rRNA* gene variants and possibly also *msp4*, *groEL* or *ankA* gene variants, may cycle differently in the blood of infected hosts, however, the epidemiological consequences of cyclic variation during persistent infection in different hosts are still unknown (Granquist et al., 2010c). The MSP4 is believed to be involved in the host-pathogen interaction and therefore may show host specific characteristics due to selective pressures exerted by the host immune systems, thus a high sequence heterogeneity is observed among *A. phagocytophilum* strains in this particular gene (Massung et al., 2003; de la Fuente et al., 2005a). Red deer for instance, previously shown to carry strains that show similarities with ovine strains in the *16S rRNA* (100%) and *ank* (99%) gene sequences (Stuen et al., 2001), have recently been shown to carry *msp4* genotypes that appear distinct from sheep variants (Stuen et al., 2013). This stands in contrast to earlier assumptions that red deer and occasionally roe deer may contribute to a natural transmission cycle in Europe, also involving livestock and humans (Alberdi et al., 2000; Rymaszewska, 2008). Characterization of variations in the *msp4* sequence, have shown similar structures of strains isolated from humans and dogs in the US (de la Fuente et al., 2005a). Homologous isolates from horse and donkey in California and Italy, respectively, and separate clustering in ruminants are additional examples of evolutionary aspects related to host susceptibility and geographical distribution of this organism (de la Fuente et al., 2005a). Similar patterns have been observed when comparing human, dog, and rodent strains with horse and ruminant strains based on components of the type IV secretion system (Al-Khedery et al., 2012). A German roe deer strain is different in the MSP4 by 23 amino acid changes, compared to the HZ-reference strain representing an outlier of the diversity within the species (de la Fuente et al., 2005a; Ladbury et al., 2008). The diversity of partial *msp4* gene in Norwegian sheep and Austrian wild ungulates have shown great variation in sequence types (Ladbury et al., 2008; Silaghi et al., 2011b), while little heterogeneity has been shown for this gene among isolates from horses (Silaghi et al., 2011b,d).

Investigations of the variable part of the *msp2 (p44)* gene have shown a clustering into variants obtained from ruminant species and those from dogs, horses, and humans, as well as a clustering into those from Europe and the US (Silaghi et al., 2011b,d).

The *ank* gene has also been used to assess the degree of phylogenetic relationship between strains of *A. phagocytophilum* as this gene is considered less conserved among strains and even more appropriate for high resolution phylogenetic studies (Massung et al., 2000; von Loewenich et al., 2003). In one study, *ankA* gene sequences were found to separate into four clearly distinct clusters. Sequences from dogs, humans, horses, and cats were found exclusively in cluster I, whereas samples from sheep, cows, European bison, and red deer were parts of clusters I and IV. Roe deer sequences were almost exclusively contained in clusters II and III. Based on these results, roe deer seems unlikely to be reservoir of human granulocytic anaplasmosis (Scharf et al., 2011), which supports the findings from studies mentioned earlier.

## Research goals and approaches

Thus far, it is not clear if the differences in infection rates in vectors and hosts outlined above truly reflect differences in vector competency of the vector species and reservoir competency of the host species or whether they reflect differences in the opportunities to acquire the infections (i.e., encounter rates). Previous studies have indicated the existence of enzootic cycles of gene variants in relation to species of ticks and hosts. The knowledge about infection cycles are important for infection and disease control in domestic animals and humans. Future studies should therefore investigate the relationship between genetic strains of *A. phagocytophilum*, ticks and different hosts, by genetic fingerprinting and blood meal analysis in order to unravel the ecology and phylogeographic distribution of *A. phagocytophilum* in nature for evidence based risk assessment and risk management. Vector competence of different tick species should be studied, especially considering the potential niche cycles and great variety of strains and variations in the different geographic areas. Which hosts and vectors that competently can keep which variants in endemic cycles in nature should be unraveled.

Further studies should investigate pathogenesis and mechanisms of persistence in host infections. The complexity of cellular and humoral immune responses in rickettsial diseases may be important targets of prophylactic and metaphylactic treatment strategies to control and cure infections by *A. phagocytophilum* in animals and humans. Factors involve in pathogenicity of the different variants should therefore be elucidated.

Cell culturing and novel molecular tools allow for rapid sequencing and annotation of whole genome structure. Several comprehensive contributions on *A. phagocytophilum* proteomics from experimental studies in culture systems, tick- and mouse models have been provided (Lin et al., 2011; Troese et al., 2011; Mastronunzio et al., 2012; Kahlon et al., 2013). However, tick and ruminant host interactions with highly pathogenic strains of the bacterium, like the Norwegian Sheep variant 1 (Stuen et al., 2002), should be studied by use of proteomic approaches to reveal key elements for future control strategies in management of this intrusive disease in livestock production. Longitudinal studies to investigate antigenic variation on genomic levels during persistent infections may reveal hitherto unknown mechanisms of immune evasion and persistence, useful in development of diagnostic and therapeutic approaches. To achieve prophylaxis by vaccination further studies on mechanisms of immune evasion and infection strategies are required. The whole genome of several variants of the bacterium has to be sequenced in order to do comparative genomics and develop proper recombinant vaccine antigens for future cross-infection studies.

### Conflict of interest statement

The authors declare that the research was conducted in the absence of any commercial or financial relationships that could be construed as a potential conflict of interest.
